# Effect of cadmium toxicity on growth, physiochemical parameters and antioxidant system of castor seedlings

**DOI:** 10.1016/j.heliyon.2024.e36536

**Published:** 2024-08-20

**Authors:** Vishal Srivashtav, Deepika Verma, Rohan Kansara, Sanjay jha, Abhinav Singh

**Affiliations:** a Plant Biotechnology Laboratory, Rajiv Gandhi South Campus, Banaras Hindu University, Mirzapur, 231001, U.P, India; b Food Quality Testing Laboratory, N.M. College of Agriculture, Navsari Agricultural University, Navsari, 396450, Gujarat, India; c Department of Plant Molecular Biology and Biotechnology, ASPEE Shakilam Agricultural Biotech Institute, Navsari Agricultural University, Athwa Farm, Surat, 395007, Gujarat, India; d Department of Agricultural Statistics, Rajiv Gandhi South Campus, Banaras Hindu University, Mirzapur, 231001, U.P, India

**Keywords:** Anatomy, Antioxidant, Biomass, Heavy metal, Metabolic, *Ricinus communis*

## Abstract

The research was aimed to determine the potential impact of cadmium contamination on *Ricinus communis*. The glucose-6-phosphate dehydrogenase (G6PDH) activity in the root was highest when exposed to 0.2 mM of Cd, with an increase of 15.63 % and 14.48 % at 0 and 24 h, respectively, compared to its control. However, citrate synthase (CS) activity declined in leaves, in contrast, to root, i.e., 12.22 % at 48 h of Cd stress. Isocitrate dehydrogenase (ICDH) activity was maximum in leaves at 0.2 mM of Cd at 0 and 24 h, i.e., 12.36 % and 13.08 % respectively, and later decreased in activity was seen in roots and leaves as the Cd stress increased. Moreover, the level of malate dehydrogenase (MDH) declined in leaves as the Cd level increased, while activity increased in roots at 0.4 mM of Cd i.e., 17.21 %, 17.52 %, and 10.53 % at 0, 24, and 48 h respectively. The important metabolite, glutathione level in the roots of SKP 84 was higher than in the leaf extract. A decline in biomass of up to 28.70 % and 30.91 % and plant length of up to 20.80 % and 26.10 % in shoot and roots, respectively, tolerance index was maximum at 0.2 mM, i.e., 98.62 % was seen. The leaves had 35.40 % catalase (CAT) activity, while the roots had 78.26 % guaiacol peroxidase (GPX) activity at 0.6 mM of Cd. At 0.2 mM of Cd, the maximum activity of ascorbate peroxidase (APX) was observed, with 67.32 % and 62.85 % activity in roots and leaves respectively. However, a reduction in the SOD activity was seen as the Cd stress increased. Increased Cd levels decreased chlorophyll but increased MDA and proline content in leaves at 0.8 mM of Cd, i.e., 82.92 % and 21.7 %, respectively. It indicated that *R. communis* SKP 84, a *fusarium* wilt resistance line, is also tolerant to Cd and can be used for phytoremediation in Cd-contaminated areas.

## 1 Introduction

It is crucial to address the issue of heavy metal pollution as it can severely impact the health and well-being of both organisms and human beings [1]. The extreme and insufficient use of pesticides and fertilizers in the soil, fixed with an increase in industrial and mining activities, are the major reasons for heavy metal contamination of water and soil. Amongst many heavy metals, Cd is the major pollutant because of its strong toxicity to ecosystems and health.

Cadmium enters the plant through roots, which are distributed to all its organs by taking over the transport pathways of micronutrients involving Fe, Mn, and Zn [2]. A negative gradient due to proton excretion across the plasma membrane of root cells operates cation absorption, including Cd^2+^ [3]. After diffusion through the apoplast, Cd^2+^ ions use transport proteins to cross the plasma membrane under −120mV membrane potential, where Cd^2+^ accumulates passively. Some of the Cd^2+^ in the cytosol diffuses to intracellular organelles through transport proteins, while other enters xylem sap by active transport where the ions ultimately form complexes with ligands which, along with the free ions, are passed to the aerial parts of the plant accompanied by xylem sap flow which was driven *via* transpiration [4]. The photosynthetic activity and rate of transpiration are altered, and changes in stomatal conductance, relative water content, and electrolyte leakage were also observed due to Cd stress [5]. Higher Cd level in plants causes phytotoxicity in plants by negatively influencing the net growth of plant height and dry weight biomass, which has been described previously in certain plants such as *Pisum sativum* [6], *Sassafras tzumu* Hemsl, [7]. Cadmium stress produces oxidative stress by releasing reactive oxygen species (ROS) in response to which the capacity of the antioxidant defense system increases to provide protection [[8], [9], [10]]. Antioxidants, namely, catalase (CAT), ascorbate peroxidase (APX), superoxide dismutase (SOD), guaiacol peroxidase (GPX), malondialdehyde content, proline content play an important function in plant defense mechanisms for abiotic stress [[8], [9], [10], [11]] and against Cd stress in *Ricinus communis* L [12]. Furthermore, it affects the metabolic enzymes deleteriously. Malate dehydrogenase (MDH), which is NAD-dependent, catalyzes the reversible conversion of malate to oxaloacetate, whereas NAD^+^-dependent isocitrate dehydrogenase (ICDH) creates 2-oxoglutarate, CO_2_, and NADH by catalyzing the oxidative decarboxylation of isocitrate yet under Cd stressed conditions and the activity of MDH and ICDH reduces [13]. Glucose-6-phosphate dehydrogenase (G6PDH) determines the operation of the pentose phosphate (PP) pathway, but its activity decreases due to Cd stress [14]. Citrate synthase (CS) condenses acetyl-CoA and oxaloacetate to create citrate in the first enzymatic step of the tricarboxylic acid (TCA) cycle. However, the studies showed citrate synthase activity declines when Cd is present [15]. Glutathione, being the precursor of phytochelatins, chelates Cd which sets the basis for an increase in rice shoot and root cellular glutathione content, resulted on exposing it to abiotic stress [16].

*Ricinus communis* L., known as Castor (Family: Euphorbiaceae), is one of the ancient, industrially important, and non-edible oil crops of the world, which shows medicinal values and economic importance. Castor is well thought out by researchers to possess great potential for heavy metal accumulation [17]. SKP 84, a castor cultivar, consists of 46.5–47.5 % of oil content, and the weight of 100 seeds ranges from 28.5 to 29.5 g [18]. The castor variety SKP 84 was utilized to create two different cross combinations. The first one, SKP 84 × JI 437, which is a female parental line, is known for its ability to produce early crops. The second combination, SKP 84 × JI244, is considered a great choice for seed production per plant, as it involves good x good combiner [19] and thus may contribute to the development of a desirable castor gene pool. Castor cultivar SKP 84, having a high rate of germination, is highly resistant to fusarium wilt, and all these can be utilized for variety production [20]. From a phytoremediation point of view, mature castor can tolerate heavy metal stress at certain concentrations due to its large biomass, while the seedlings may not [21]. Consequently, it's important to assess the amount of tolerance and accumulation of Cd in SKP 84 at the seedling stage. To obtain more information about the effects of cadmium distribution at different cellular levels and at different stages in SKP 84, the antioxidant system also showed a response to Cd stress, indicating a biochemical mechanism at play. Further research is needed to fully understand the effects of cadmium on this variety of castor and to develop strategies to mitigate its impact. As there are no such reports of Cd stress in castor with response to its changes in the level of metabolic enzymes were assessed. So, there is a need to exploit the metabolic pathways and their correlation with the antioxidant defense system and morphological changes in castor var SKP 84.

## 2 Materials and methods

### 2.1 Sources and preparation of seeds

**Fig. 1:**
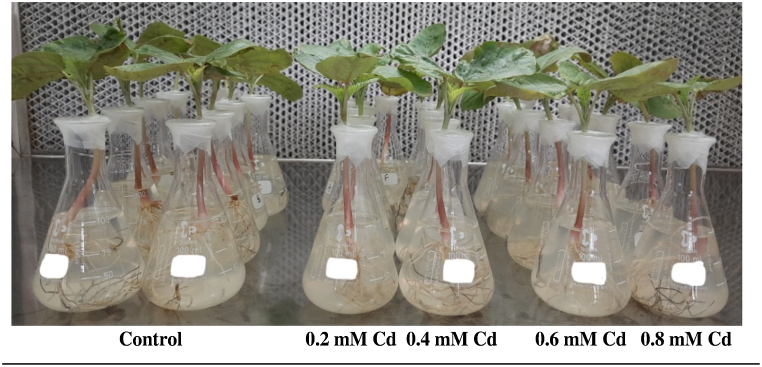
Supplementation of castor cultivar SKP 84 grown hydroponically with different concentrations of CdCl2.

Seeds of fusarium wilt-resistant parental line SKP 84 were acquired from the Castor-Mustard Research Station, Sardarkrushinagar Dantiwada Agricultural University, Gujarat, India. Seeds of castor variety SKP 84 were sanitized with 70 % alcohol with Tween 20 for 10 min followed by HgCl_2_ for 4 min and washed thoroughly using distilled water. After the imbibitions, a single seed in each test tube was planted containing full strength of (MS) medium [22]. A mixture of vitamins consisting namely, 0.2 mg of pyridoxine, 0.4 mg of thiamine, 0.4 mg of nicotinic acid and 100 mg of myoinositol is added to MS basal salts medium (pH 5.8) along with 15 g of sucrose. After that the inoculated seeds were incubated at 25 ± 1 °C for 14/10 h (dark/light) with a photon flux density of 100 μmol photon m^−2^s^−1^ till two leaf stages. Then, seedlings were treated with MS media with 0.2, 0.4, 0.6, and 0.8 mM of CdCl_2_ for 0, 24, and 48 h respectively for metabolic enzymes assay (Fig. 1). However, the seedling was maintained for 14 days under these conditions for antioxidant and metabolites assay. Control seedlings were kept in MS media without CdCl_2_. Then, the castor leaf and root samples were transferred to liquid nitrogen and kept there at −70 °C until the study's variables are measured.

Three separate stages of the biochemical parameters analysis were performed: (i) the control without CdCl_2_, (ii) 24 h after CdCl_2_ treatment, and (iii) 48 h after CdCl_2_ treatment. For antioxidants, enzymes, and metabolites analysis was done after 14 days of treatment with various CdCl_2_ concentrations. Fresh leaf and root of castor with Cd stress at 0, 0.2, 0.4, 0.6, and 0.8 mM at 0, 24, and 48 h a total of 15 samples with 3 replications were gathered, washed with tap water, and then rinsed with Milli Q water before being subjected to biochemical analysis.

### 2.2 Metabolic enzyme assay

The plant material was pulverized using a pre-chilled mortar and pestle. Subsequently, it was combined with a 0.05 mM phosphate buffer solution having a pH of 7.5 and added 250 mg of polyvinyl poly pyrrolidone (PVP). After centrifuging of mixture for 10 min at 10,000×*g* in 4 °C, the supernatant was assayed at 25 °C in a 1.0 ml reaction. Activities of the enzymes-glucose-6-phosphate dehydrogenase (G6PDH), malate dehydrogenase (MDH), citrate synthetase (CS), and isocitrate dehydrogenase (ICDH) can be observed in the resulting supernatants as well as protein content was determined.

A spectrophotometric approach was employed to determine the malate dehydrogenase activity (NAD^+^-MDH, EC 1.1.1.37) based on the rate of oxidation at 340 nm. Enzymes required to cause the oxidation of 1 μmol of NADH in 1 min at 25 °C was measured as an enzyme activity unit. A final volume of 1 ml of the reaction mixture containing 20 mM MgCl_2_, 0.5 mM oxaloacetate, 1 mM EDTA, and 0.1 mM NADH in a buffer of 100 mM Tris-HCl (pH-7.8) was used to assess the activity of MDH in the reaction. Variants without oxaloacetate served as control [23].

The G6PDH (G6PDH, EC 1.1.1.49) assay was done at room temperature by monitoring the decrease of NADP level at 340 nm. At pH 8.1, the molar absorbance of NADP was calculated to be 17.8 mM^−1^ cm^−1^. In cuvette, reaction mixture of 1 ml contains 50 mM of Tris-Cl (pH 7.8), 0.2 mM of glucose-6-phosphate, 0.2 mM of 6-phosphogluconate, 1 mM of MgCl_2_, 0.1 mM of NADP and an aliquot of enzyme extracts (0.1 ml) [24].

The activity of citrate synthase (CS, 4.1.3.7) was determined by observing the changes in 5, 5-dithiobis (2-nitrobenzoic acid) (DTNB) at 412 nm. In 1.0 ml of assay mixture, the following components were present: Cell lysate, DTNB, 1 mM, acetyl-CoA, 0.2 mM, oxaloacetate, 0.2 mM, and tris-HCl (pH = 8.0), 100 mM. The reaction was in progress by adding oxaloacetate. At 412 nm, the molar absorbance coefficient was determined to be 13.6 mM^−1^ cm^−1^. The CS activity was calculated using the rate of rise in absorbance [25].

The activity of ICDH (1.1.1.42) was assessed by monitoring the production of NADPH at 340 nm. A 1 ml of the reaction mixture containing 100 mM of Tris-HCl (pH 7.5); 5 mM of MnCl_2_; 10 mM of NADH; 10 mM of isocitrate; and 20 % of glycerol. The NADP's molar absorbance coefficient was determined to be 17.8 mM^−1^ cm^−1^ at 260 nm [26].

### 2.3 Growth parameters and tolerance index

Plant samples were collected by clipping the shoot at the culture level after 14 days. The roots and aerials were cleaned four or five times in distilled water after being washed in a weak detergent solution. Plant parts were kept for 48 h of oven drying at 70 °C. The samples' total dry weight was measured by electronic balance. The biomass of Cd-treated castor compared to its control plant was measured by the tolerance index (TI). TI=Wcd/WControl, Where, Wcd(g) = biomass after Cd treatment, Wcontrol(g) = biomass of control plant.

### 2.4 Antioxidant enzymes assay

Root and leaf extracts of SKP 84 were prepared by homogenizing 0.05 g sample in 0.5 ml extraction buffer for analyzing antioxidant enzymes activity viz. ascorbate peroxidase (APX), guaiacol peroxidase (GPX), catalase (CAT), and superoxide dismutase (SOD) where extraction buffer was composed of 1 mM EDTA and 1 % polyvinylpyrrolidone (PVP) in buffer with 50 mM of sodium phosphate buffer (pH 7.4). The mixtures were then centrifuged at 10,000 rpm for 20 min and the supernatant was utilized during enzyme assay [27]. Enzymatic activities like CAT, SOD, GPX, and APX were assayed.

The amount of H_2_O_2_ was used to calculate the catalase activity (EC 1.11.1.6) oxidized in μMol/min/g protein by observing the decline in 240 nm absorption. The 3 ml mixture for spectrophotometric analysis was comprised of 50 μl enzyme extract, 500 μl of 10 mM H_2_O_2_, and 2.45 ml of 50 mM sodium phosphate buffer (pH 7.0) [28].

The 3 ml reaction mixture for spectrophotometric assay and the total SOD (EC 1.15.11) activity was analyzed by adding 1.6 ml of 50 mM of sodium phosphate buffer (pH 7.8), 250 μl of 2 mM riboflavin, 250 μl of 75 μM NBT, 500 μl of 13 mM methionine, 300 μl of 0.1 mM EDTA and 100 μl enzymes in test tubes where riboflavin was finally introduced. Shake the test tubes and be subjected to fluorescence by a 15-W lamp, as a light source. The reaction takes place for 10 min and photo reduction of NBT was quantified as a rise in absorbance at 560 nm. Blank and control were both executed in the same mode but lacked illumination and enzyme extract respectively [29].

The GPX (EC 1.11.1.7) activity was performed spectrophotometrically at 470 nm in homogenates by measuring absorption due to formation of tetra guaiacol in the reaction comprised 1.65 ml of sodium phosphate buffer (pH 7.0) 50 mM, 300 μl of 0.1 mM EDTA, 50 μl enzyme extract, 500 μl of 10 mM guaiacol, and 500 μl of 10 mM H_2_O_2_ [30].

The APX (EC 1.11.1.11) activity was measured instantly in a 3 ml reaction that includes 500 μl of 0.1 mM ascorbic acid, 300 μl of 0.1 mM of EDTA, 500 μl of 0.1 mM H_2_O_2_ and 0.1 ml of fresh enzymes in a solution containing 1.6 ml of 50 mM sodium phosphate (pH 7.0). The absorbance level was taken at 290 nm based on the oxidation of ascorbate [30].

### 2.5 Determination of metabolites

#### 2.5.1 Malondialdehyde content

Malondialdehyde concentration was measured by TBARS assay. 0.1 g of leaf and root samples was homogenized in 0.1 % of TCA in 5 ml and had been centrifuged at 3500×*g* for 20 min. A 1 ml of supernatant was separated to which 20 % TCA (4 ml) with 0.5 % (w/v) TBA was supplemented as well as the mixture was kept at 95 °C for 30 min. Subsequently, the mixture was cooled on ice before being centrifuged at 10,000×*g* for 10 min. Both 532 nm and 600 nm were used to measure absorbance [31].

#### 2.5.2 Proline content

Leaf and root samples of 0.5 g were homogenized in 10 ml of 3 % of sulphosalicylic acid and centrifuged at 3500×*g* for 10 min. A total 2 ml of supernatant and 2 ml of acid ninhydrin (consisting of 30 ml of glacial acetic acid, 1.25 g of ninhydrin, and 20 ml of 6 M phosphoric acid) were added. After 1 h keeping in a boiling water bath, the mixture was cooled. Toluene (4 ml) was added and mixed by vortexing. Reading was recorded at 520 nm by taking the toluene layer [32].

#### 2.5.3 Glutathione (GSH) detection

Glutathione was detected using a glutathione quantification kit (Dojindo, Japan). Firstly, add 20 μl of coenzyme solution to each well, 120 μl of Buffer solution, and 20 μl of enzyme. Then the plate was incubated at 37 °C for 5 min. Add 20 μl of GSH standard and the sample solutions. After 10 min of incubation at 37 °C, add 20 μl of substrate i.e., DTNB (5, 5′-dithiobis (2-nitrobenzoic acid)), working solution, and incubate the plate for 10 min at room temperature. The absorbance at 415 nm was measured using a microplate reader.

### 2.6 Anatomical studies

For shoot and root anatomy, 14 days old seedlings were examined. The transverse section of shoot and root of castor variety SKP 84 were safranin-stained and mounted in 20 % glycerin. A compound light microscope was used to examine the transverse section of the stem and leaf. (Magnus Trinocular microscope Model: CH-20i TR. LED) at 40× magnification. A Micrograph of the section was taken using a digital camera attached to the microscope.

### 2.7 Statistical analysis

The metabolic enzymes, metabolites and ROS-related enzymes data were analyzed by using SPSS 16.0 with the use of analysis of variance (ANOVA). For normality and homogeneity of variance Shapiro Wilk and Bartell's test respectively has performed. Therefore, on the basis of p-value (>0.05) we found that the data is normal and variances among groups are not significant difference. The Data is presented as mean value ± SE and compared using Tukey's test. Standard error was calculated by using the mean value from three replications.

### 2.8 Results and discussion

The plant tri-carboxylic acid (TCA) system is involved in the mitochondrial respiratory system, which is connected with malate and pyruvate. However, NADPH produced during the TCA cycle is a key component to having scavenging systems of H_2_O_2_. Due to the stress caused by Cd, the activity of dehydrogenase synthase enzymes is observed, eventually leading to the generation of enough NADPH supply. This, in turn, affects the antioxidant activity in the matrix.

### 2.9 Effect of Cd on metabolic enzymes

**Fig. 2:**
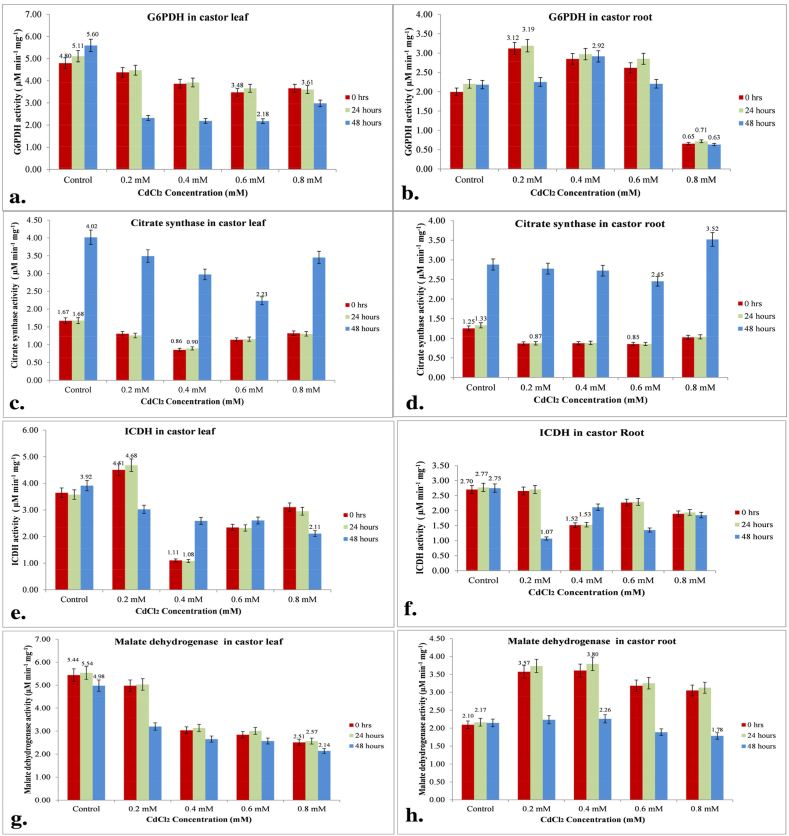
Effect of different concentrations of CdCl2 on metabolic enzyme activities. The castor seedlings were cultured in MS medium with different Cd concentrations at 24 and 48 h. The activities of metabolic enzymes **(a)** G6PDH leaf **(b)** G6PDH root **(c)** CS leaf **(d)** CS root **(e)** ICDH leaf **(f)** ICDH root **(g)** MDH leaf **(h)** MDH root.

Glucose-6-phosphate dehydrogenase (G6PDH) is the rate-limiting enzyme responsible for the generation of NADPH in the PP pathway by regulating glucose metabolism [33,34]. To determine whether G6PDH plays any role in Cd stress tolerance, we measured the activity of G6PDH in castor at various concentrations of Cd. There was a significant decrease in G6PDH activity in castor leaf after exposure to various concentrations of CdCl_2_. At 0 h, the activity decreased by 8.55 %, 19.32 %, 27.50 %, and 23.62 % for 0.2, 0.4, 0.6, and 0.8 mM concentrations, respectively, compared to the control untreated plant. This trend continued at 24 h, with a decrease of 12.42 %, 23.22 %, 28.29 %, and 2.43 %, and at 48 h, with a significant decrease of 58.49 %, 61.00 %, 61.18 %, and 46.69 %, respectively. This may be attributed to the stress caused by Cd, which affects the activity of dehydrogenase synthase enzymes and ultimately the antioxidant activity in the matrix (Fig. 2a). While, in castor roots, enhancement of G6PDH activity was observed till 0.6 mM of Cd stress and maximum activity was found at 0.2 mM of Cd at 0 and 24 h, i.e., 15.63 % and 14.48 % and 0.4 mM of Cd at 48 h, i.e., 13.35 % as compared to its control. In contrast, a significant decrease in G6PDH activity in roots was measured at 0.8 mM of Cd at 48 h was 71.18 %. After 48 h of treatment, there was a noticeable increase in activity up to 0.4 mM, which was 1.34 folds higher than the untreated control plants. However, the activity levels then started to decline (Fig. 2b). A similar observation has been made for G6PDH in the root of wheat whose transcript accumulation after 2 h of NaCl treatment activity was higher at 12 h (2.2 folds) and subsequently declined after 48 h of treatment [35]. Plant responses to Cd stress may be mediated by the rapid induction of G6PDH, which may also facilitate Cd response by supplying precursors/cofactors for related biosynthetic pathways. The PP pathway's NADPH production is a component that limits acetyl-CoA incorporation into fatty acids, which is important for lipid synthesis.

The conversion of intermediates, citrate, and isocitrate is crucial for various metabolic pathways. They aid in reductant reaction and contribute to fatty acid synthesis, which supports the glyoxylate cycle. These intermediates are also significant for the TCA cycle, an important process for energy production, and gluconeogenesis, which produces glucose from non-carbohydrate sources. The reaction catalyzed by these enzymes may be a critical end to TCA regulation [36,37]. The first enzyme synthesizes citric acid through a condensation reaction by acetyl-CoA. The second enzyme synthesizes 2-oxoglutarate from isocitrate by reversible isomerization and conversion of the oxidized and reduced form of glutathione. The concentration of intermediates, their distribution, and transportation to various cell compartments due to Cd stress can have a significant impact on cell metabolism in castor plants. In particular, there was a noticeable increase in CS activity in both the leaves and roots of the SKP 84 variety of castor plants, with levels being much higher in plants treated for 48 h compared to those treated for 0 or 24 h. This suggests that Cd stress can greatly affect the metabolic processes of castor plants. Similarly, the amount of citrate secreted throughout 24 h also increased at 0.05 mM Al treatment for 3 h when exposed to light in the root of soybean [38]. Citrate synthase activity was appreciably decreased in 0, 24, and 48 h Cd treated castor leaf in SKP 84, maximum depletion of CS was found at 0.4 mM of Cd which was 48.83 % and 46.69 % at 0 and 24 h while at 48 h maximum activity was lost at 0.6 mM of Cd i.e., 44.4 % than that of its control untreated plant (Fig. 2c). As in leaves, a similar pattern of activity was observed in roots also which shows a maximum decrease of 31.76 %, 35.74 % and 14.82 % at 0, 24 and 48 h respectively at 0.6 mM of Cd treated plants but there was an increase of 12.22 % was observed at 48 h of 0.8 mM of Cd treated plant (Fig. 2d). In some case of roots where exhaustion of malate and citrate concentration occurred at 24 h of Aluminum (Al) treatment in Jiyu 62 genotypes as compared to its control (0 h) due to lower citrate efflux [39]. Under Cd stress, malate decreased first, followed by citrate in the SKP 84 genotype of castor.

Dehydrogenases are the key enzymes to generate energy which occurs to regulate metabolic activities like fatty acids and amino acid biosynthesis [40]. Based on the research, it appears that the regulation of the TCA cycle is influenced by certain metabolic enzymes including ICDH, MDH, and fumarase. To determine the impact of Cd stress, it is necessary to observe the activity of these enzymes. The study found that in castor leaf, the activity of ICDH increased steadily at 0.2 mM of Cd, with a 12.36 % and 13.08 % increase at 0 and 24 h, respectively. However, after 48 h, there was a reduction of up to 22.80 % compared to the untreated control plant (Fig. 2e). Similarly, the ratio of NADPH/NADP+ was significantly increased due to oxidative stress (1 mM H_2_O_2_) in *Cryptococcus neoformans,* an intracellular fungus in *idp1*Δ strain contrasted with its wild type [41]. In contrast, the activity of ICDH in root saw a tremendous decline at 0.4 mM of Cd i.e., 43.95 % and 44.84 % at 0 and 24 h as compared to its control plants while at 48 h, a decrease of 61.07 % was seen (Fig. 2f). These findings suggest that Cd stress can potentially impact the regulation of the TCA cycle through the activity of metabolic enzymes such as ICDH.

The MDH is responsible for forming malate from oxaloacetate utilizing NADPH to generate NADP^+^, an electron acceptor [42]. Cd-induced altered metabolic enzyme behaviour is supposed to contribute to decreased MDH activity in plants treated with Cd. The MDH activity gradually declined in the leaves of castor seedlings at 0, 24, and 48 h of Cd-treated plants compared to its untreated control plant as the Cd concentrations increased. There was a consistent pattern where the highest activity was observed at 24 h. This trend was almost identical across all concentrations. The study found that the activity of MDH was significantly reduced in the castor leaves of SKP 84. The most significant decrease was observed in plants treated with 0.8 mM of Cd at 0, 24, and 48 h, which showed a decline of 53.77 %, 53.60 %, and 56.97 % respectively, as compared to the untreated control plants (Fig. 2g). The activity of aminating (NAD + -MDH) increased gradually in roots and the maximum was at 0.4 mM of Cd in the medium i.e., around 17.21 %, 17.52 %, and 10.53 % at 0, 24, and 48 h respectively, as compared with its control and beyond that at 0.6 mM and 0.8 mM of Cd after 48 h a concentration-dependent gradual inhibition of enzyme activity was observed (Fig. 2h). Similarly, a gradual inhibition in the MDH activity under *invitro* conditions was recorded due to NaCl treated rice plant range from 1 to 1000 mM in a concentration-dependent manner [43].

### 2.10 Growth of castor cultivar

A hydroponic experiment indicated that increasing Cd concentrations reduced castor cultivar biomass. After treating with 0.2 mM, 0.4 mM, 0.6 mM, and 0.8 mM of Cd, the root biomass of SKP 84 declined by 2.72 %, 11.81 %, 14.54 %, and 30.91 %, respectively, while the shoot biomass decreased by 1.0 %, 6.72 %, 14.06 %, and 28.7 % than that of control. Similarly, the tomatoes experienced a significant decrease in dry weight, with reductions of up to 75.60 % and 74.07 % being observed as a result of Cd stress [44]. The plant tolerance index (TI) decreased in a dose-dependent manner and was observed maximum (98.62 %) in seedlings treated with 0.2 mM compared to other Cd treatments. The results indicate that Cd stress inhibited the seedling's growth at higher concentrations (0.6 mM and 0.8 mM). Our results align with previous investigations reported in soybean seedlings, where the biomass of roots was reduced by 32 % and 47 %, while the shoot biomass was reduced by 21 % and 29 % on the 10th day of 50 μM and 100 μM Cd stress [45]. The length of a plant's root and shoot of castor seedlings decreased as the Cd concentrations increased. This is an important consideration for anyone interested in the growth and development of plants, especially in areas where Cd contamination is a concern. After subjecting the plant to different levels of Cd stress, it was observed that the decrease in root length ranged from 9.1 % to 28.40 % in comparison with the control plant. Similarly, the decrease in shoot length varied from 1.09 % to 20.8 %. The degree of reduction in both root and shoot length was directly proportional to the concentration of Cd stress applied, with the highest concentration of 0.8 mM causing the most significant reduction in both root and shoot length. (Table 1). Growth measures such as cell division and elongation are inhibited due to heavy metal toxicity through its effect on the proton pump and membrane potential [44]. In contrast, plant height is increased due to ZnO and Fe nanoparticles of up to 37 and 35 % in wheat [46]. Earlier studies also reported a decrease in root and shoot lengths with increasing Cd concentration up to 27.7–50.0 % and 24.3–36.6 %, respectively, in S2-4 and 16–024 castor varieties after five days of Cd exposure [12].

**Table 1:** Effect of Cadmium on biomass, plant length and tolerance index of castor bean cultivar SKP 84.

Cadmium Treatment	Biomass (g/plant)		Plant length (cm)		Tolerance Index (%)
	Shoot	Root	Shoot	Root	
**Control**	0.349 ± 0.03	0.109 ± 0.02	18.20 ± 2.00	8.80 ± 1.80	
**0.2 mM**	0.334 ± 0.02	0.103 ± 0.04	18.00 ± 1.95	8.00 ± 1.43	98.62
**0.4 mM**	0.305 ± 0.02	0.088 ± 0.03	17.80 ± 1.52	7.90 ± 1.41	91.99
**0.6 mM**	0.281 ± 0.01	0.080 ± 0.03	15.00 ± 2.26	6.65 ± 0.46	85.81
**0.8 mM**	0.230 ± 0.01	0.074 ± 0.03	14.40 ± 1.91	6.30 ± 0.25	70.70

Values are shown as a mean ± SE (n = 3).

### 2.11 Effect of Cd on antioxidant enzymes

Under hydroponic conditions, Cd treatment changed the antioxidant enzyme activities and levels of metabolites, including malondialdehyde, proline, and chlorophyll. These findings are crucial for future research in this area.

**Fig. 3:**
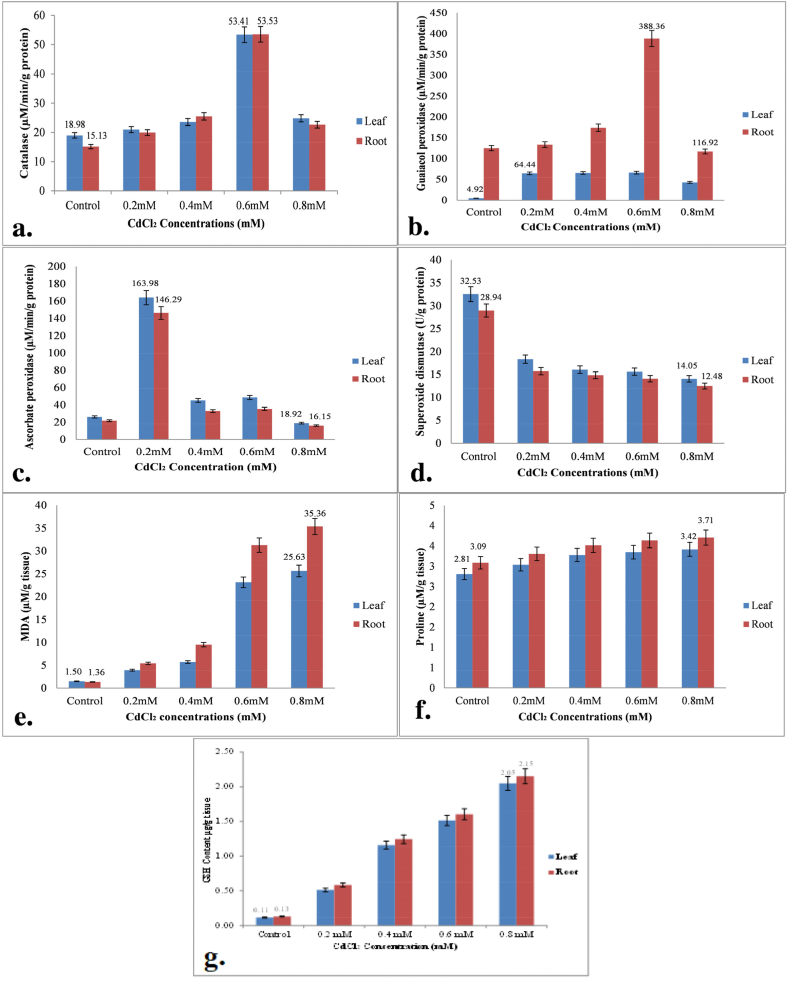
Effect of different concentrations of CdCl2 on ROS content antioxidant enzyme activities, MDA and Proline content in leaves and roots of SKP 84 castor seedlings. The seedlings were grown for 14 days of hydroponically in half MS medium with different Cd concentrations (0.2, 0.4, 0.6 and 0.8 mM). **(a)** Catalase **(b)** GPOX **(c)** APOX **(d)** SOD **(e)** MDA content **(f)** Proline content **(g)** GSH content.

After subjecting SKP 84 to varying concentrations of Cd, it was observed that both the roots and leaves of the plant displayed an increase in catalase activity. The maximum activity was recorded in seedlings treated with 0.6 mM Cd, where the activity was found to be 28.14 % and 35.40 % higher in both roots and leaves, respectively, compared to control seedlings. However, the decrease in activity at 0.8 mM of Cd (Fig. 3a) is probably due to the accelerated rate of ROS generation and enzyme inhibition which results from the binding of Cd to the thiol group of the enzyme causing protein disruption. It's interesting to note that found similar results in their study with *B. juncea* leaves, showing a significant increase in catalase activity under Cd stress in a concentration-dependent manner [47]. This increase in catalase activity is likely an adaptive response by SKP 84 seedlings to the Cd stress, helping to prevent tissue damage caused by oxidative stress. The increased catalase activity is possibly the adaptive response by SKP 84 seedlings to the Cd stress to surmount tissue damage by oxidative stress. A similar trend was maintained in GPX activity which increased with an increase in Cd stress and then decreased at 0.8 mM in both roots and leaves of castor seedlings. At 0.6 mM of Cd stress, the activity of GPX showed a significant increase of 78.26 % in roots and 31.07 % in leaves, as compared to the control. This increase in GPX activity could be attributed to the plant's defensive response against excessive free radicals in roots (Fig. 3b). Similarly, it has reported increased GPX activity in *Lemna gibba* and *Lemna minor* with increased Cd concentrations after 4 days of Cd exposure [48]. An increase in GPX activity is involved in lignin biosynthesis which is responsible for Cd binding to the cell wall. The activity of Ascorbate peroxidase (APX) was higher in the roots than in the leaves of castor seedlings of SKP 84. It's interesting to note that the activity initially increased and then gradually declined in both the roots and leaves, and this trend was dose-dependent. These observations suggest that APX plays a crucial role in regulating the oxidative stress response in castor seedlings, and its activity is closely linked to the dose of the stressor. There was a tremendous increase in APX activity initially at 0.2 mM of Cd stress i.e., 67.32 % in the roots and 62.85 % in leaves of seedlings as that of its control plants which declined further with an increase in Cd concentration (Fig. 3c). The decreased APX activity at higher Cd concentrations because of H_2_O_2_ and its derivatives are more likely to form and may be due to lack of Fe as a cofactor in APX metalloprotein complex. As per previous studies, it has been observed that APX activity is more prominent in the roots than in the leaves at higher concentrations of Cd. Prior research also showed similar results where APX activity was found more in the roots rather than the leaves which dropped at higher Cd concentrations i.e. 2.2 μM and 4.7 μM in Adamello and Ofanto cultivars of wheat, respectively, following ten days of Cd treatment [49]. The SOD activity in the roots and leaves of the seedlings gradually decreased as the Cd level increased in comparison to its control. The study found that the activity of SOD (superoxide dismutase) was significantly reduced in both the leaves and roots of the plants as the concentration of Cd (cadmium) increased. Specifically, there was a gradual decrease in SOD activity by 43.65 %, 50.63 %, 51.98 %, and 56.80 % in the leaves, and 45.68 %, 48.75 %, 51.31 %, and 56.87 % in the roots, compared to the control. This suggests that exposure to Cd can negatively impact the antioxidant defense system of plants (Fig. 3d). Decrease in SOD activity may be due to H_2_O_2_ produced from Cd stress in various cells during enzymatic and non-enzymatic processes. Our results align with the investigation done, where they showed reduced SOD activity in *Dittrichia viscosa* treated with 5, 10, and 15 mg Cd/l over 10 days in hydroponics than control that inferred higher oxidative stress [50]. Although, studies have suggested that the enzymes such as catalase and SOD get inactivated in the formation of the Cd complex with various ligands, like sulfur in cysteine, which is highly stable, thereby, increasing the oxidative damage in plants, Cd's impact on SOD activity is still in the debate which probably is dependent on plant species or varieties, Cd exposure time and its concentration in the medium [51].

### 2.12 Malondialdehyde (MDA) and proline content

Malondialdehyde (MDA) is a byproduct of lipid peroxidation that accumulates in plants by exposing them to oxidative stress. It is a cytotoxic compound whose level increased as Cd stress increased in SKP 84 in comparison to control (Fig. 3e). At higher Cd concentrations (0.8 mM), there was a significant increase in MDA levels observed in both the roots and leaves, with an increase of approximately 74.00 % and 82.92 %, respectively. This could be attributed to low levels of SOD which were unable to efficiently scavenge ROS, leading to damage of the cell membranes. The previous experiment had also described alike results where MDA content increased in wheat significantly, after Cd treatment i.e., 3.68 fold as compared to its control plant, however, the exogenous application of melatonin, sodium nitroprusside, and sodium hydrosulphide reduced the level of MDA content [52]. Proline content of Cd-treated seedlings under hydroponic conditions steadily increased as the level of Cd increased. It was found that proline accumulated more in the roots and leaves of castor plants when exposed to 0.8 mM of Cd. In fact, the accumulation was about 20.06 % and 21.7 % higher than that of the control group respectively (Fig. 3f). It is a metabolite and shows more accumulation due to Cd exposure and played a significant role in osmoregulation and osmotolerance. Similarly, it has also reported higher proline accumulation which was about 10 folds of control in the leaves and roots of *R. communis* when given Cd treatment for 45 days that forms a non-toxic Cd-proline complex by chelating Cd ions [53]. Here, the Cd induces more ROS accumulation due to interference in the physiological processes of the castor. To protect against ROS various organelles show an antioxidant defense system by producing antioxidant defense system enzymes and antioxidant compounds.

### 2.13 GSH content

Apparently, exposure to Cd has been shown to promote the synthesis of glutathione, which is indicated by the GSH level. This could have important implications for plant growth and health, especially since we know that increasing the concentration of Cd can have negative effects on biomass, plant height, and tolerance index. In the control, GSH was detected and quantified in leaves and root of SKP 84 which shows an increase in GSH content as the Cd concentration increases. Upon conducting tests on SKP 84, it was found that the root had a substantially higher GSH level than the leaf (P < 0.05) following exposure to 0.2, 0.4, 0.6, and 0.8 mM of Cd. The highest GSH level was observed at 0.8 mM, with an increase of 82.1 % and 83.33 % in the leaves and roots, respectively, as compared to the control (Fig. 3g). These results suggest that the root of SKP 84 possesses a stronger ability to synthesize thiol-rich peptides than the leaves and could be regarded as a Cd detoxification inducer. Furthermore, an increase in GSH is vital for Cd detoxification and is also the substrate for phytochelatins forming a complex with Cd and sequestering into a vacuole [16]. Similarly, the exogenous application of GSH leads to increased growth, photosynthesis, biomass, and improved antioxidant activity due to Cd stress was reported [54].

### 2.14 Anatomical changes of the root and shoot cells in Cd treatment

**Fig. 4:**
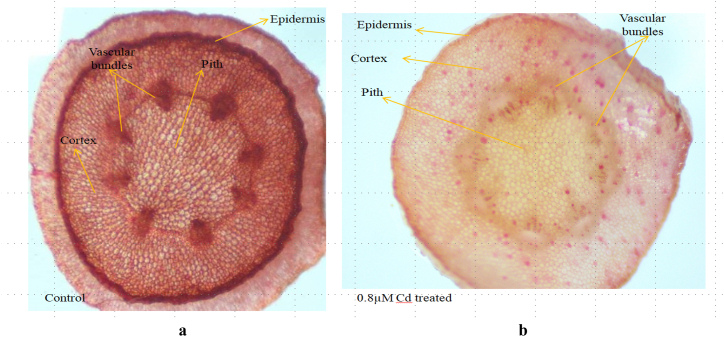
Transverse Section of castor bean shoot. **a**: Control; **b**: 0.8 μM Cd treated.

**Fig. 5:**
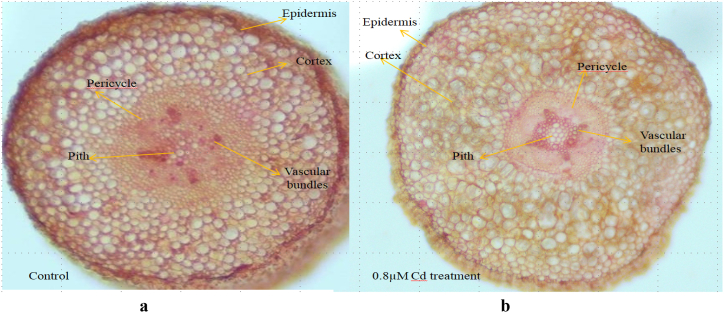
Transverse section of castor bean root. **a**: Control; **b**: 0.8 μM Cd treated.

The decrease in both root and shoot diameter observed in the plant treated with Cd (0.8 mM) may be attributed to the reduction in cell size and vascular element. This is in contrast to the control group, which did not show any significant changes in diameter. The epidermal cell was much thicker in the Cd-treated plant shoot than in the control (Fig. 4a and b). However there was not much damage seen in the epidermal and cortical cells of cd treated root cells than that of the Cd treated shoot cells (Fig. 5a and b). The result indicates that the root cell of castor SKP 84 possesses more Cd detoxification than shoot cells.

## 3 Conclusion

Based on our findings, increasing the concentration of Cd resulted in a decrease in biomass, plant height, and tolerance index. This emphasizes the need to monitor Cd levels in the soil for optimal plant growth. There appears to be a limit to the amount of Cd that plants can handle. We also observed a rise in catalase and GPOX activity up to 0.6 mM of Cd, followed by a decline. However, decreases in SOD activity while, initially, APOX activity first increased, then it started to fall. As the Cd stress rises, an increase in MDA, Proline, and GSH content was observed. The G6PDH activity was increased initially in the root, but later decreased. Citrate synthase activity declined in leaves and roots while higher at 48 h after Cd stress, similar trends were seen in ICDH activity in roots and leaves. The activity of MDH was gradually decreased in leaves however; activity increased up to 0.4 mM of Cd, and later decreased.

## Ethical approval

Not applicable.

## Consent to participate

Not applicable.

## Consent to publish

Not applicable.

## Data availability statement

The data that support the findings of this study are available on request.

## CRediT authorship contribution statement

**Vishal Srivashtav:** Writing – original draft, Visualization, Validation, Resources, Methodology, Investigation, Formal analysis, Data curation, Conceptualization. **Deepika Verma:** Writing – review & editing, Resources, Project administration, Methodology, Investigation, Conceptualization. **Rohan Kansara:** Methodology, Data curation, Conceptualization. **Sanjay jha:** Writing – review & editing, Validation, Methodology, Investigation, Formal analysis. **Abhinav Singh:** Writing – review & editing, Visualization, Validation, Software.

## Declaration of competing interest

The authors declare that they have no known competing financial interests or personal relationships that could have appeared to influence the work reported in this paper.
